# Supplementary material for: Organic Acid-Catalyzed Subcritical Water Hydrolysis of Immature Citrus unshiu Pomace
Source: Foods. 2021 Dec 22;11(1):18. doi: 10.3390/foods11010018 (PMC8750635; doi:10.3390/foods11010018)
Supplement: Supplementary file 1 [file foods-11-00018-s001.zip › foods-1495356-supplementary.pdf]

Supplementary Materials

Table S1. Preliminary experimental result: hydrolysis yields of flavonoid monoglucosides and aglycons from immature citrus pomace with different organic acids.

**Flavonoid**	**Hydrolysis yield (mg/g dry sample)**						
	**Citric**	**Ascorbic**	**Formic**	**Acetic**	**Tartaric**	**Malic**	**Succinic**
Hesperidin	18.67	28.45	18.99	29.19	11.90	18.94	28.70
Hesperetin-7-O-glycoside (H7G)	13.73	9.02	12.11	7.04	13.00	11.93	8.92
Hesperetin (HT)	6.09	3.66	4.45	2.30	5.25	4.02	2.69
Narirutin	4.17	7.34	4.07	7.53	2.50	4.13	7.05
Prunin (PR)	3.92	2.62	3.35	2.13	3.52	3.28	2.61
Naringenin (NG)	1.67	0.85	1.18	0.49	1.44	1.03	0.60
Total hydrolysis products	25.41	16.15	21.09	11.96	23.21	20.26	14.82

Total hydrolysis products: H7G+PR+ HT+NG

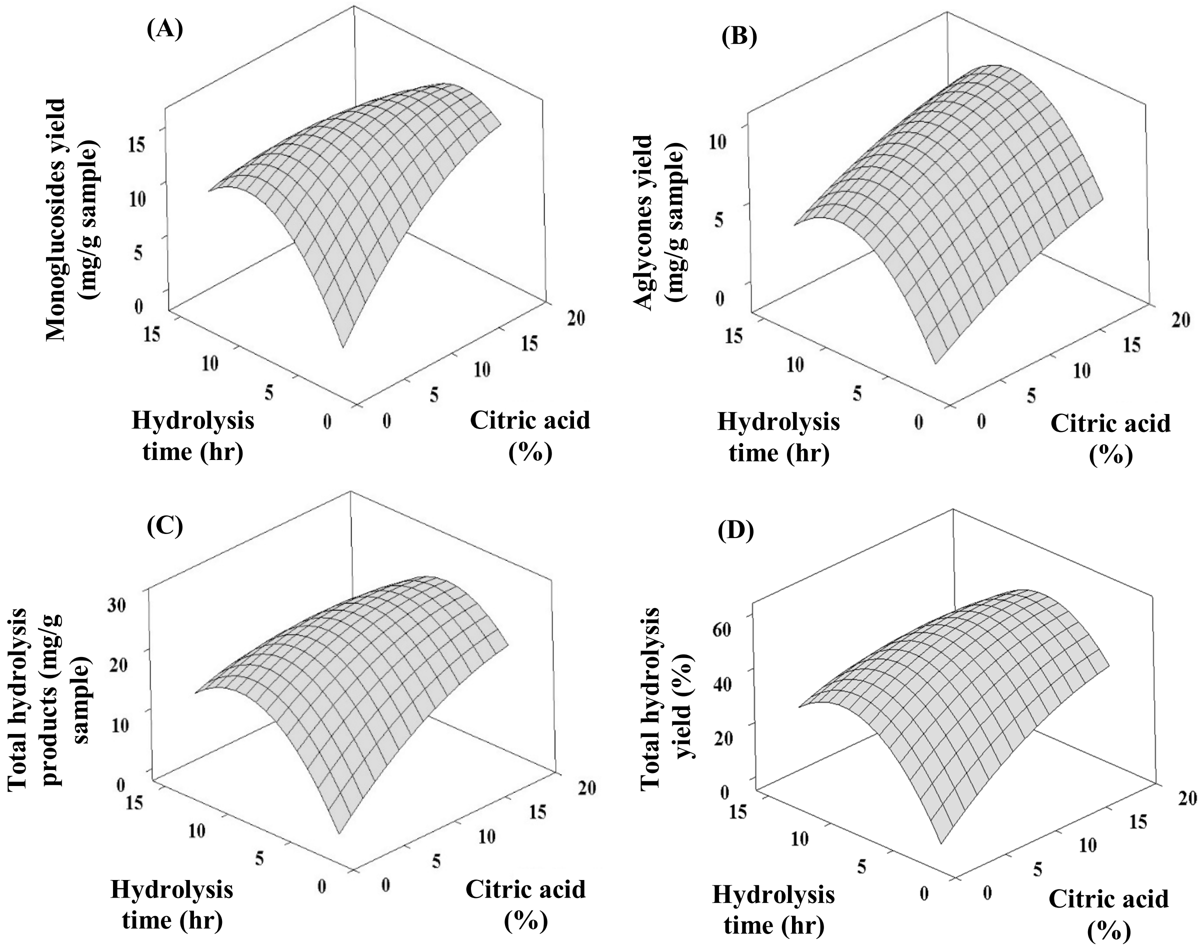

Fig. S1. Three-dimensional response surface plots for (A) flavonoid monoglucosides, (B) aglycons, (C) total hydrolysis products, and (D) % total hydrolysis yield.
